# Osteochondral Tissue Engineering *In Vivo*: A Comparative Study Using Layered Silk Fibroin Scaffolds from Mulberry and Nonmulberry Silkworms

**DOI:** 10.1371/journal.pone.0080004

**Published:** 2013-11-19

**Authors:** Sushmita Saha, Banani Kundu, Jennifer Kirkham, David Wood, Subhas C. Kundu, Xuebin B. Yang

**Affiliations:** 1 Biomaterials and Tissue Engineering Group, School of Dentistry, University of Leeds, Leeds, United Kingdom; 2 Department of Biotechnology, Indian Institute of Technology, Kharagpur, India; 3 Biomineralisation Group, School of Dentistry, University of Leeds, Leeds, United Kingdom; Osaka University, Japan

## Abstract

The ability to treat osteochondral defects is a major clinical need. Existing polymer systems cannot address the simultaneous requirements of regenerating bone and cartilage tissues together. The challenge still lies on how to improve the integration of newly formed tissue with the surrounding tissues and the cartilage-bone interface. This study investigated the potential use of different silk fibroin scaffolds: mulberry (*Bombyx mori*) and non-mulberry (*Antheraea mylitta*) for osteochondral regeneration *in vitro* and *in vivo*. After 4 to 8 weeks of *in vitro* culture in chondro- or osteo-inductive media, non-mulberry constructs pre-seeded with human bone marrow stromal cells exhibited prominent areas of the neo tissue containing chondrocyte-like cells, whereas mulberry constructs pre-seeded with human bone marrow stromal cells formed bone-like nodules. *In vivo* investigation demonstrated neo-osteochondral tissue formed on cell-free multi-layer silk scaffolds absorbed with transforming growth factor beta 3 or recombinant human bone morphogenetic protein-2. Good bio-integration was observed between native and neo-tissue within the osteochondrol defect in patellar grooves of Wistar rats. The *in vivo* neo-matrix formed comprised of a mixture of collagen and glycosaminoglycans except in mulberry silk without growth factors, where a predominantly collagenous matrix was observed. Immunohistochemical assay showed stronger staining of type I and type II collagen in the constructs of mulberry and non-mulberry scaffolds with growth factors. The study opens up a new avenue of using inter-species silk fibroin blended or multi-layered scaffolds of a combination of mulberry and non-mulberry origin for the regeneration of osteochondral defects.

## Introduction

Osteochondral defects (OCDs) result from traumatic injuries or natural degradation of cartilaginous tissue with aging and encompass serious damage to articular cartilage and/or underlying calcified subchondral bone [1]. Therapeutic approaches for OCDs include allografts, stimulation of bone marrow and debridement [2]. Allografts are associated with the risk of immune rejection or disease transmission [3], while bone marrow stimulation treatments are only palliative and not completely curative [4].

Tissue engineering provides a promising approach for the treatment of osteochondral defects employing biomaterials, progenitor cells and growth factors [5]. Attempts have been made with Ostecel (hydroxyapatite and autologous MSCs), INFUSE™ (recombinant human bone morphogenetic protein), VITOSS® (calcium phosphate-bone bonding protein), CORTOSS (synthetic bone void filler) [6] and TruFit (PLGA Plug) [7] as scaffolding materials to regenerate osteo/chondral tissues. Most of these materials are limited for applications in long term sustained tissue regeneration due to their synthetic origin nature, potential to raise inflammatory or foreign body responses in host systems and inconsistent/unacceptable degradation rates accompanying neo-tissue formation [7]–[10]. Silk protein fibroin, is a natural material that possesses excellent biocompatibility [11], [12] with less toxic degradation products [13] and an absence of adverse immune responses within host systems. Moreover, slow and controllable biodegradability, robust mechanical properties, plasticity in water based processing into diverse pore sizes and porosity based on tissue specific requirements, along with ease in incorporation and stabilization of bioactive molecules in contrast to other currently available biomaterials makes it an ideal scaffold for use in regenerative medicine [14]. Silk is also abundant as a raw material in nature. Other biomaterials exploited as scaffolds, so far, do not offer similar extent of advantages. Silk scaffolds, which acts as a template in regenerative therapeutics for a wide range of tissues, is FDA approved for ligament and tendon repair and commercially marketed by Serica [11].

Growth factors, especially those belonging to the super family of transforming growth factor beta (TGF-β) play multifunctional roles in the context of tissue engineering. TGF-β is involved in chondroinduction both *in vitro* and *in vivo* [15]. Bone morphogenetic proteins (BMPs), especially BMP-2, have proven osteoinductive capacity promoting osteogenic differentiation [16]. BMP-2 has also been FDA approved for treating long bone fractures. However, the combinational effects of TGF-β3 and BMP-2 with silk scaffolds on osteochondral reconstruction *in vivo* still remain elusive.

While silk has been used as a scaffold for cartilage and/or bone tissue regeneration individually, the materials' ability to support osteochondral tissue regeneration to our knowledge has never been evaluated *in vivo*. Osteochondral tissue regeneration is more complex than individual osseous or chondral tissue regeneration as the scaffold involved needs to support cartilage repair and the chondral interface needs to be supported by underlying subchondral bone *in situ* [17]. The use of silk scaffolds for regeneration of either cartilage or bone has been well documented in previous in vitro and in vivo studies. However, reports of the use silk fibroin for complete regeneration of osteochondral defects *in vivo* are sparse [18]. In addition, to our knowledge, a comparative study of mulberry silk of Bombyx mori and non-mulberry silk of Antheraea mylitta for *in vivo* osteochondral repair is novel. The aim of the present study was to evaluate the osteo- and/or chondro-inductive ability of silk (mulberry and non-mulberry origin) fibroin biomaterials using human bone marrow stromal cells (hBMSCs) *in vitro*. Silk fibroin scaffolds from mulberry and non-mulberry origin differ in their inherent natural properties and thus comparison of two kinds of silk scaffolds is important. The investigation is further expanded to growth factor guided (TGF-β3 or BMP-2) *in vivo* cellular infiltration and neo-matrix formation on multi-layered cell-free (acellular) silk scaffolds (experimental group) in order to demonstrate the feasibility of using two different silk scaffolds as three dimensional (3D) implantable platform in osteochondral therapeutics.

## Materials and Methods

The human bone marrow stromal cells (hBMSCs) were purchased from Lonza Ltd (USA). All animal studies were carried out under the PI's UK Home Office Project License approval (PPL:40/3361).

### Fabrication of silk scaffolds

Aqueous silk protein fibroin solutions of *Bombyx mori* (Bm) and *Antheraea mylitta* (Am) were prepared following the standard protocol described elsewhere [19]. Briefly, fibroin from Bm was obtained from cocoons by a multistep process. The cut pieces of cocoons were boiled in 0.02 M Na_2_CO_3_ for 1 hr to remove completely the highly hydrophilic protein sericin [20]. We got about 21% of sericin for the bivoltine (two crops in a year) species of Bm. The degummed fibroin fibers were then washed thoroughly in running water overnight and dried in laminar hood. Finally, these fibers were solubilized in 9.3 M lithium bromide (LiBr) to yield Bm fibroin in solution. The fibroin solution is dialyzed using 12 kDa dialysis tube by changing water for several times with continuous stirring. The silk fibroin of Am was obtained directly from silk glands of the mature 5^th^ instar larvae. The silkworms were dissected to obtain the silk glands. The glands were washed thoroughly in de-ionized water to remove the traces of water soluble sericin. The gel like hydrophobic fibroin was squeezed out in SDS-Tris buffer system to dissolve the Am fibroin [19]–[20]. The fibroin solution was then dialyzed extensively against de-ionized water to remove the traces of the respective solvent system. The aquous silk fibroin solutions were then cast into appropriate moulds (5 mm diameter ×2 mm thickness) and lyophilized to obtain 3D silk scaffolds. The process incorporated a slow and even pre-cooling at −20°C to obtain uniform porous interconnected structures with defined pore sizes.

### Scanning electron microscopy

The porous nature of the silk scaffolds was examined using SEM (JEOL JSM 5800) after gold coating. The pore sizes were determined by calculating random 26 pores from SEM image using ImageJ 1.40 g software [21].

### Porosity measurement

Porosity of the scaffolds was calculated by solvent displacement method using hexane, an inert non-solvent for silk as described elsewhere [21]. Briefly, the scaffolds were immersed in known volume of hexane (V_1_) in a graduated cylinder for 5 min. Hexane permeated through the interconnective pores of the scaffolds causing negligible swelling or shrinkage. After 5 min, the total volume of hexane-impregnated scaffolds with hexane (V_2_) and only the residual hexane volume (V_3_) were recorded. The porosity (Є) was calculated as follows: 
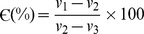



### Isolation and Cultivation of hBMSCs

Human bone marrow stromal cells (hBMSCs) from hematologically normal donors (Lonza, USA) were cultivated in α-MEM containing 10% (v/v) FCS. Passage 4 (P4) cells were used for all of the in *vitro* experiments.

### Assessment of in vitro chondrogenic and osteogenic differentiation

Equal numbers of BMSCs (2.5×10^5^ cells/scaffolds) were seeded dynamically onto silk scaffolds following sterilization as described previously for 24 hrs. and maintained under static culture conditions at 37°C and 5% CO_2_ [19]. Constructs (scaffolds with cells) were then divided into two groups and cultured with chondro-inductive medium (α-MEM supplemented with 10 ng/mL TGF-β3 (Peprotech, USA), 10^−8^ M dexamethasone, 100 µM ascorbate-2-phosphate and 1× insulin transferring selenium (ITS) [22]) for 4 weeks or osteo-inductive medium (α-MEM containing 10^−8^ M dexamethasone and 100 µM ascorbate-2-phosphate) [23] for 8 weeks at 37°C and 5% CO_2_.

### Image analysis

After 4 weeks for chondroinduced constructs (n = 4) and 8 weeks for osteoinduced constructs (n = 4), cells were labeled with Cell Tracker™ Green 5-chloromethylfluorescein diacetate (Invitrogen, USA) and Ethidium homodimer-1 (Molecular Probes, USA) [24] and viewed using confocal laser scanning microscopy (Leica, Germany). Cell adhesion and viability for all constructs were examined and compared by three blind assessors.

### Histological analysis

Histological staining was carried out as described elsewhere [24], [25]. Briefly, the constructs after CLSM analysis were fixed in 10% neutral buffered formaldehyde, embedded in paraffin wax and 5 µm thick serial sections were prepared using a microtome (Leica). The sections were stained with Alcian Blue [0.5% (w/v)] and Sirius Red [0.3% (w/v)]. The stained sections were imaged under an Olympus, BX50 microscope and analyzed using NIS Elements BR software (Ver. 3.0). Cellular differentiation for all constructs were examined and compared by blind assessors.

### Creation of osteochondral defects and scaffold placement

All *in vivo* studies were carried out under UK Home Office Project License approval (PPL:40/3361). () Osteochondral defects (1.8 mm diameter × 1 mm depth) were created in the patellar groove of the knee joints of male Wistar rats (230–250 g) using a trephine burr. Animals were divided into five groups listed in table 1 (n = 4 defects per group). For groups EG2 and EG4, two scaffold discs of each silk type (mulberry and non-mulberry) were coated with BMP-2 (0.1 µg/µL) and another scaffold coated with TGF-β3 (1 ng/µL) was placed on the top and glued together using fibrin glue (Baxter, Austria). These composites were used to seal the defects. For groups EG1 and EG3 scaffolds of each silk type without any growth factor treatment were used. The control group (CG) was left empty.

**Table 1:** Silk fibroin scaffold groups information for *in vivo* module.

	Experimental Group 1 (EG 1)	Experimental Group 2 (EG 2)	Experimental Group 3 (EG 3)	Experimental Group 4 (EG 4)	Control Group (CG)
**Layer 1**	Mulberry silk	Mulberry silk + TGF-β	Non-Mulberry silk	Non-Mulberry silk + TGF-β	Empty defect
**Layer 2**	Mulberry silk	Mulberry silk + BMP-2	Non-Mulberry silk	Non-Mulberry silk + BMP-2	Empty defect
**Layer 3**	Mulberry silk	Mulberry silk + BMP-2	Non-Mulberry silk	Non-Mulberry silk + BMP-2	Empty defect

Different combination of layered scaffold with/without growth factors was used to repair osteochondral defects in rat patella femoral grooves.

### Immunohistochemical staining

After 8 weeks, staining with specific antibodies against type I collagen (Mouse monoclonal anti-collagen I; 1∶300; Abcam), and type II collagen (Mouse monoclonal anti-collagen II; 1∶600; Calbiochem) was performed on 5 µm thick formalin-fixed paraffin-embedded tissue. Endogenous peroxidase activity was blocked using 2% hydrogen peroxide and an enzymatic antigen retrieval step was carried out using chymotrypsin (Sigma). Samples were incubated with the primary antibody (overnight at 4°C). Staining was achieved using peroxidase conjugated secondary antibodies (EnVision Kit, Dako) and visualized using an Olympus BX50 microscope. Nuclei were counter stained with hematoxylin.

## Results

The use of silk scaffolds for regeneration of either cartilage or bone has been well documented in previous *in vitro* and *in vivo* studies. However, report of the use silk fibroin for complete regeneration of osteochondral defects *in vivo* is sparse [18]. In addition, to our knowledge, a comparative study of mulberry silk of *Bombyx mori* and non-mulberry silk of *Antheraea mylitta* for *in vivo* osteochondral repair is novel.

### Micro-architecture of 3D silk fibroin network

**Figure 1:**
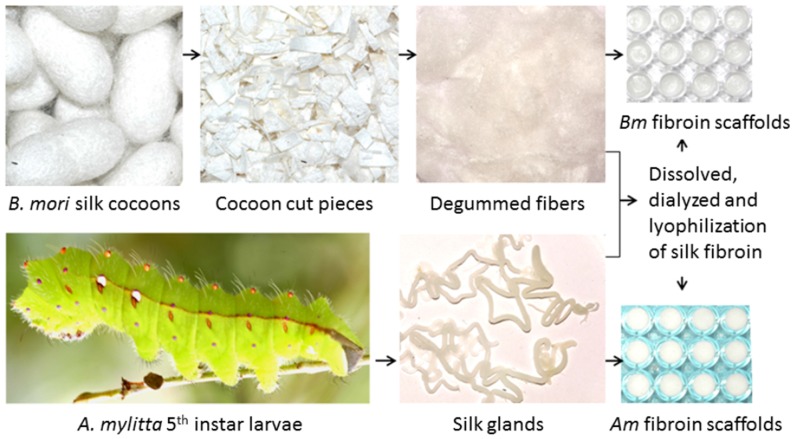
Schematic representation of fabrication of 3D scaffolds of *B. mori* and *A. mylitta*. Cut pieces of *B. mori* silk cocoons were alkaline hydrolyzed, degummed fibers dissolved and dialyzed to yield silk fibroin solution. The mature 5^th^ instar larvae of *A. mylitta* were dissected to isolate silk glands and dialyzed to obtain gland silk fibroin solutions. The solutions were used to fabricate 3D scaffolds.

**Figure 2:**
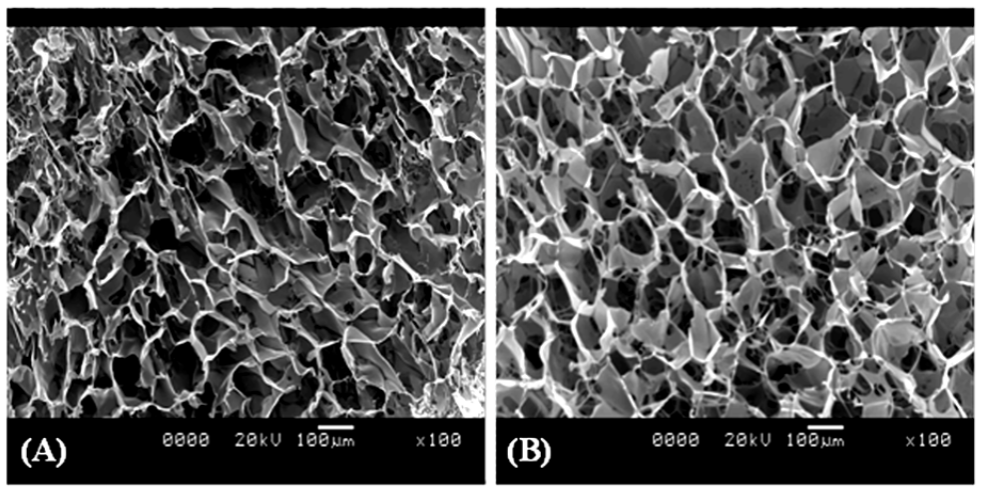
Scanning electron micrographs revealing the pore micro-architectures and interconnectivity within 3D silk fibroin scaffolds. (A) *B. mori*; (B) *A. mylitta*.

Silk fibroin scaffolds prepared by lyophilization (Figure 1) demonstrated a porous and open-ended pore micro-architecture at almost all surfaces with suitable interconnectivity (Figure 2). Based upon SEM images, these pores varied according to whether the scaffolds were *Am* or *Bm*; pores with more open, interconnected and well defined boundaries were observed in *Am*, in contrast to *Bm*. Average pore sizes (based on the measurement of random 26 pores) of *Bm* and *Am* 3D porous networks were 72±10 µm (60–96 µm) and 74±10 µm (63–99 µm) respectively. In addition, porosity of the scaffolds ranged between 74 and 82% with a maximum pore size of 82±10 µm in *Am* scaffolds. The scaffolds were readily equilibrated in culture medium without any need for pre-wetting treatments.

### Cell viability and cellular morphology within 3D scaffold

**Figure 3:**
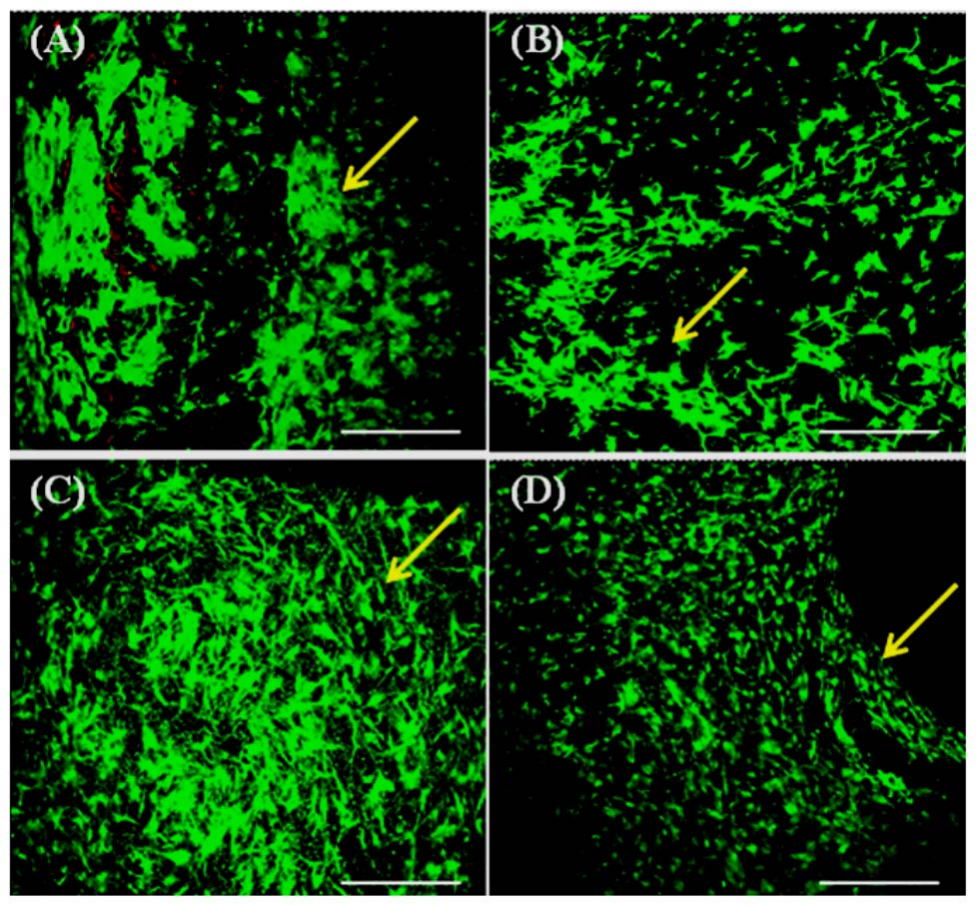
Confocal images of hBMSCs seeded on 3D silk fibroin scaffolds and cultured in chondroinductive and osteoinductive media. Non-mulberry *A. mylitta* silk constructs: (A) 4 weeks after chondrogenic culture and (C) 8 weeks after osteogenic culture. Mulberry *B. mori* silk constructs: (B) 4 weeks after chondrogenic culture; (D) 8 weeks after osteogenic culture condition. Yellow arrows indicate attachment of viable cells onto all available areas of the scaffold. Scale bars represent 300 µm.

The effect of dynamic seeding of hBMSCs on silk scaffolds was evaluated using confocal laser scanning microscopy (CLSM). 3D reconstruction using a series of CLSM slices enabled to observe cell ingrowth and spreading at different depths of the scaffolds. The micrographs visually revealed a high proportion of viable cells (green) in both scaffold groups *in vitro* (n = 4 for each group) (Figure 3). After 4 weeks in chondrogenic medium (Figure 3A & 3B) and 8 weeks in osteogenic medium culture (Figure 3C & 3D), the cells appeared spindle shaped and well distributed all over the scaffold. Three blind assessors independently assessed the extent of cell adhesion and cell viability based upon reconstructed 3D CLSM micrographs provided. The de-coded results showed that non-mulberry constructs supported higher cell numbers under chondro and osteo-inductive culture conditions compared to mulberry constructs. Both mulberry and non-mulberry constructs cultured in osteoinductive medium appeared to possess a greater number of cells than their corresponding constructs cultured under chondroinductive conditions, most likely due to the prolonged culture period of 8 weeks. Dynamic seeding with cell suspensions into the porous 3D network of polymeric scaffolds has been documented to result in rapid imbibitions and homogenous distribution of cells within the scaffolds with minimal shear stress on the cells [26]. The spindle shape of cells adhered on to mulberry and non-mulberry scaffolds along with high cellular viability observed within the scaffolds in this study indicated a permissive environment for differentiation [27]–[29].

### Histology and immunohistochemistry of in vitro constructs

**Figure 4:**
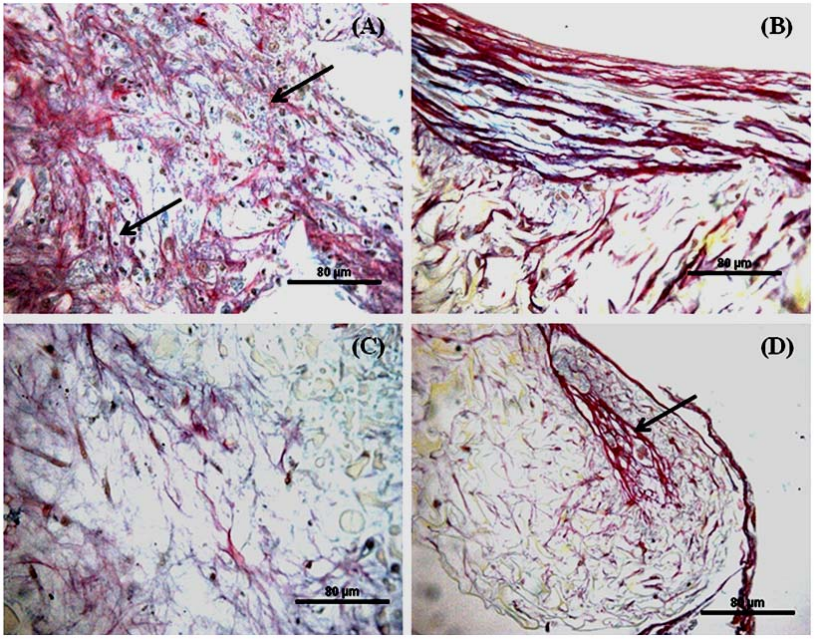
Histological appearance of constructs following *in vitro* culture. Constructs cultured in chondrogenic media for 4 weeks: (A) Non-mulberry *Am* scaffolds; (B) Mulberry *Bm* scaffolds. Constructs treated with osteogenic media for 8 weeks: (C) Non-mulberry *Am* scaffolds; (D) Mulberry *Bm* scaffolds. Scale bars represent 80 µm.

Alcian blue (AB)/Sirius red (SR) staining revealed a mixed extracellular matrix (ECM) commensurate with fibrous collagen and glycosaminoglycans (GAGs) in all groups (Figure 4). Chondrocyte like cells were enclosed in lacunae (indicated by arrows) of non-mulberry constructs cultured in chondroinductive medium (Figure 4A). The ECM formed in chondro-induced mulberry constructs exhibited a SR-collagen staining pattern typical of cartilage tissue; parallel fibres on the surface edge and perpendicular fibrillar alignment towards the interior of the tissue (Figure 4B). The typical chondrocytic morphology (round shape) and enhanced production of ECM within non-mulberry silk constructs *in vitro* are strongly suggestive of the fact that the hBMSCs had undergone chondrogenesis [28]. Prominent formation of bone nodule like structures (indicated by arrow, Figure 4D) was observed in mulberry osteo-inductive constructs and was absent in corresponding non-mulberry osteoinductive constructs (Figure 4C).

### Histology and immunohistochemistry of in vivo constructs

**Figure 5:**
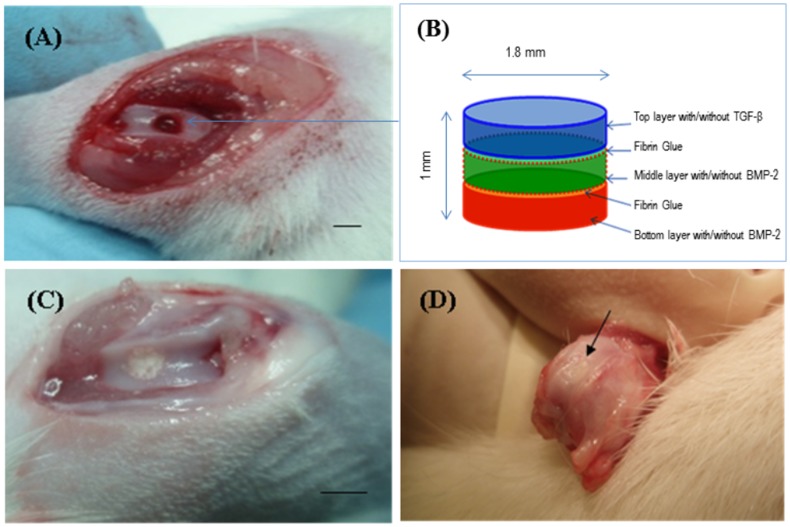
Repair of osteochondral defect in rat patella-femoral groove. (A) 1.8 mm wide and 1 mm deep osteochondral defects created using a trephine burr. (B) The multi-layered silk fibroin based scaffold with/without growth factors was prepared using 3 scaffold discs by stacking one on top of each other. (C) Scaffolds were implanted to fill the osteochondral defect. (D) After 8 weeks *in vivo*, the appearance of the repair of osteochondral defect and the interface with surrounding normal cartilage (arrow). Scale bars represented 2 mm.

**Figure 6:**
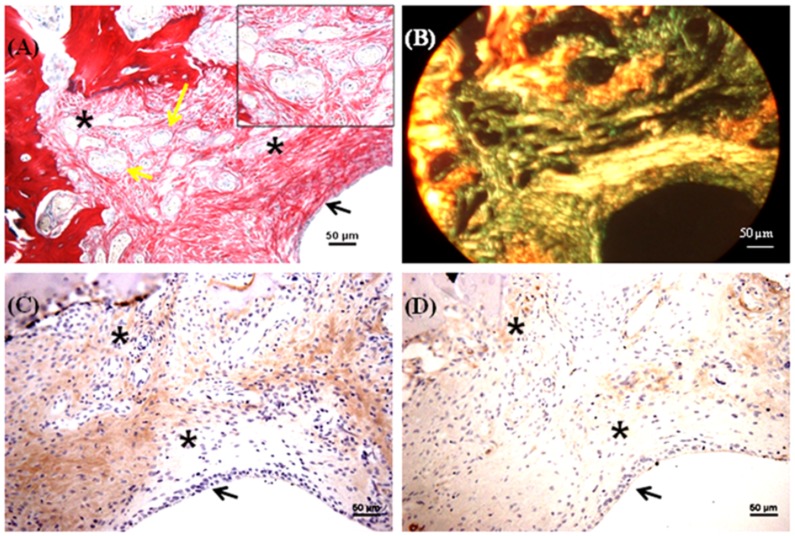
Histological and immunohistochemical appearance of osteochondral defects filled with mulberry silk scaffold (*Bm*) without growth factors *in vivo*. (A) AB/SR staining; (B) Birefringence of AB/SR section; (C) immunostaining with type I collagen and (D) immunostaining with type II collagen. Black asterisks denote the scaffold within the defects. Scale bars represented 50 µm.

**Figure 7:**
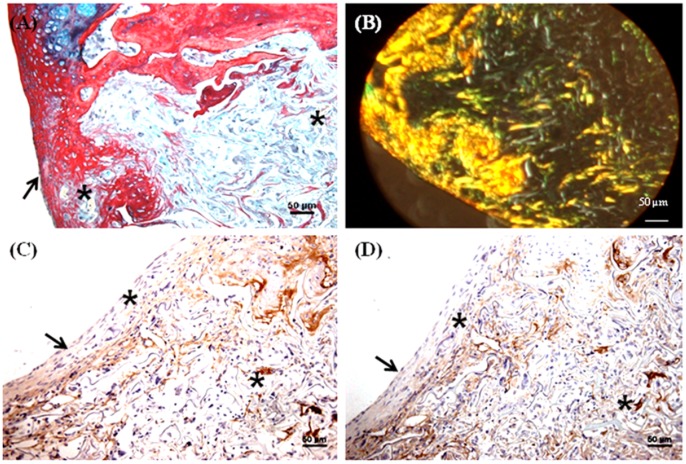
Histological and immunohistochemical appearance of osteochondral defect filled with non-mulberry silk scaffolds (*Am*) without growth factors *in vivo*. (A) AB/SR staining; (B) Birefringence of AB/SR section; (C) immunostaining with type I collagen and (D) immunostaining with type II collagen. Black asterisks denote the scaffold within the defects. Scale bars represented 50 µm.

**Figure 8:**
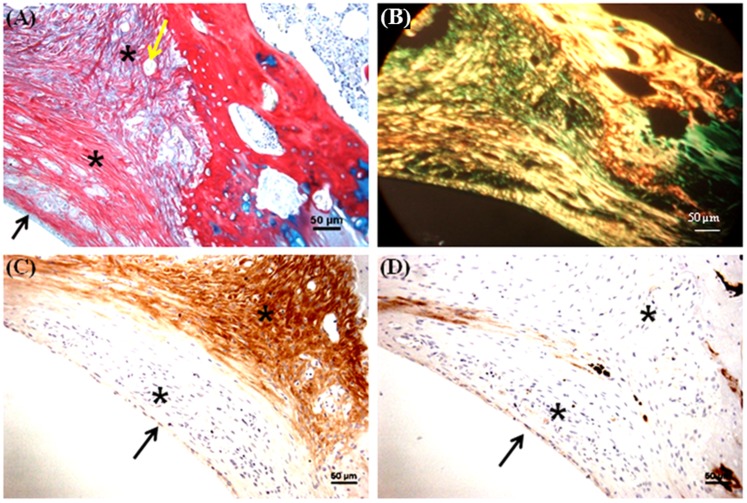
Histological and immunohistochemical appearance of osteochondral defect filled with mulberry silk scaffolds (*Bm*) treated with growth factors *in vivo*. (A) AB/SR staining; (B) Birefringence of AB/SR section; (C) immunostaining with type I collagen and (D) immunostaining with type II collagen. Black asterisks denote the scaffold within the defects. Scale bars represented 50 µm.

**Figure 9:**
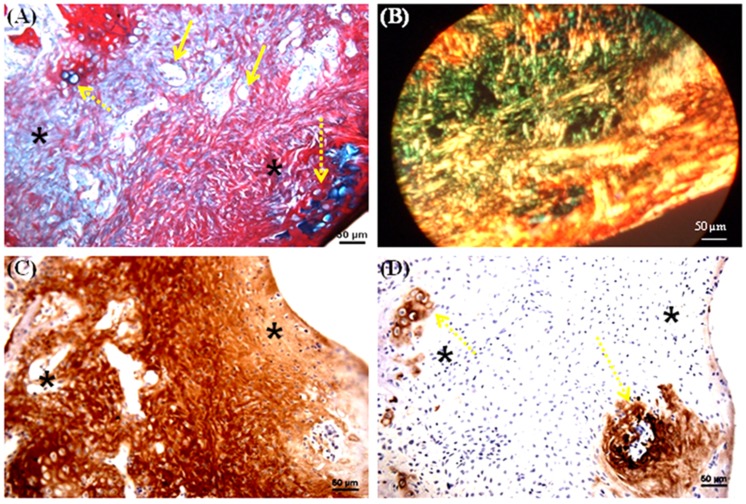
Histological and immunohistochemical appearance of osteochondral defect filled with non-mulberry silk scaffolds (*Am*) treated with growth factors *in vivo*. (A) AB/SR staining; (B) Birefringence of AB/SR section; (C) immunostaining with type I collagen and (D) immunostaining with type II collagen. Black asterisks denote the scaffold within the defects. Scale bars represented 50 µm.

A trephine was used to create the 1.8 mm diameter and 1 mm depth osteochondral defects in the patellar groove of the knee joints of Wistar rats (Figure 5A). The defects were then filled with multilayered mulberry or non-mulberry scaffold discs with or without growth factors (TGF-β and BMP-2) (Figure 5B & C). The different groups investigated are listed in Table 1. Markedly, the boundaries of the defects were detectable in all the rats treated with scaffolds after 8 weeks (Figure 5 D). Macroscopically, the defect areas were covered by smooth, glistening white neo-tissue in all constructs (Figure 5D, indicated by arrow). Microscopically, excellent integration of the neo-tissue with the host tissue was seen in all constructs with no visible cracks or fissures, which are often seen in OCD repaired with polymers [29]. The cells in experimental group, EG1 (mulberry silk without growth factors) (Figure 6) and EG3 (non-mulberry silk without growth factors) (Figure 7) had no consistent morphology compared to those within the EG2 (mulberry silk with growth factors) (Figure 8) and EG4 (non-mulberry silk without growth factors) constructs (Figure 9). ECM was significantly stained by the characteristic red of SR, indicating the presence of collagen enriched neo-matrix (Figure 6A, 7A, 8A and 9A). The positive AB staining (Figure 7A, 8A and 9A) was consistent with accumulation of GAGs in all of the implants except those of the EG1. In EG1 constructs (Figure 6A), predominant formation of collagen (SR staining) was observed throughout (from top to the base of the defect; indicated by black arrow) the construct compared to corresponding non-mulberry control constructs (EG3), where SR staining was primarily seen at the top surface of the defect (indicated by black arrow) (Figure 7A). The middle and bottom parts (towards the deep zone of cartilage and subchondal bone) of the defects of EG3 constructs exhibited ECM rich in GAGs (Figure 7A). Highly aligned collagen fibers, which were parallel at the top and perpendicular to surface of the defect from middle to base (indicated by black arrow) were observed in EG2 constructs (Figure 8A). Interestingly, islands of chondrocyte like cells at the surface and base of the defect (indicated by yellow dotted lines) (Figure 9A) were seen in EG4 constructs only. Birefringence images of AB/SR staining for all *in vivo* experimental constructs re-confirmed the histological findings (Figure 6B, 7B, 8B and 9B). The formation of blood vessels (indicated by yellow arrows) at the base of the defects were observed in all of the experimental constructs (Figure 6A, 7A, 8A and 9A). Empty osteochondral defects (control group- CG) failed to heal after 8 weeks *in vivo*, with the defect remaining clearly visible and not fully filled with ECM. Unlike all the experimental constructs which, with or without growth factors revealed the presence of a distinctive layer of cells formed at the top (i.e. close to the surface) indicating closing of the defect. This layer of cells was absent in the empty control (data not shown).

Following immunohistochemical staining of the *in vivo* experimental constructs for collagens (types I and II), strong staining of type I collagen was observed throughout the ECM of all of the defects with few areas that were non positive (Figures 6C, 7C, 8C & 9C). Both mulberry (EG1 and EG2) constructs exhibited faintly positive type II collagen staining (Figure 6D & 8D). In contrast, non-mulberry constructs (EG3 and EG4) demonstrated significant positive staining for type II collagen (Figure 7D & 9D). Specifically in the EG4 constructs, cartilage like tissues which were visible following AB staining, stained intensely for type II collagen with a higher proportion of the positive staining concentrated towards the top surface of the defect (Figure 9D). Chondrocyte like cells that appeared to be larger in size were positive for type II collagen and perhaps indicated the process of endochondral ossification towards the base of the defect (Figure 9D).

## Discussion

The successful regeneration of osteochondral defects collectively requires suitable cell sources, favorable 3D scaffolds and bio-inductive culture environments [30]. Undifferentiated bone marrow MSCs possess the potentiality to differentiate down multiple lineages including osteoblasts and chondrocytes [31]. The major limitation of using cell suspensions directly in osteochondral repair is the requirement for a large starting cell number and the possibility of cells migrating away from the recipient site [32]. Applying a scaffold based cell delivery system results in a more convenient and predictable way of controlling cell numbers and delivery to the target site. This study investigated the possibility of using silk-based biomaterials as osteochondral scaffolds and to determine their innate ability to home and differentiate endogenous progenitor cells.

The pore sizes of scaffolds significantly affects the differentiation of cells, thereby influencing the type of matrix laid and ultimately tissue formed [18], [33]–[35]. It has previously been shown that scaffolds with a pore size <150 µm were best suited for chondrogenic differentiation [34]. It has also been documented that smaller pore sizes (<100 µm) helped to induce osteochondral formation and were better suited for hypoxic conditions. The open interconnected porous architecture of silk scaffolds in the present study was supportive of ECM formation allowing adequate nutrient diffusion and neo-vascularization [36]. Thus, given that the measured pore sizes for both groups of scaffolds was less than 100 µm, it was hypothesized based on previous literature cited that the scaffolds would be suitable for osteochondral tissue regeneration. The natural presence of the integrin binding tri-peptide sequence (Arg-Gly-Asp) in *A. mylitta* fibroin [19], [37] has added an advantage to cell attachment, better cellular interaction and tissue regeneration. Further, using a slow freezing technique (−20°C) for 24 hrs, resulted in a range of pore sizes and geometry [21] perfectly mimicking the features of anatomical trabecular bone structure, which has a porosity ranging from 50% to 90% [38].

The similar pore sizes of both types of silk scaffolds used in the present study eliminated this as a potential confounder, when investigating the ability of mulberry and non-mulberry silk scaffolds to induce/support osteo/chondrogenesis with the appropriate signaling cues. hBMSCs appeared to preferentially differentiate down the chondrogenic pathway on non-mulberry silk scaffolds whilst preferential osteogenic differentiation of hBMSCs was observed on mulberry silk scaffolds *in vitro*. The overall quality of tissue generated *in vitro* by these scaffold types might be compromised by mass transfer in the static cultivation environment and may not be a true reflection of *in vivo* tissue regeneration. Thus, it was essential to compare the two scaffold types in a moderate load bearing *in vivo* environment (i.e. the patellar groove of the knee joint).

The strategic employment of growth factors in the repair of osteochondral defects [39]–[41] can lead to the functionalization of scaffolds. In the current study, this was achieved by binding growth factors using opposing gradients for TGF-β and BMP-2 respectively, i.e. for any one defect, TGF-β concentration would be highest at one end of the scaffold, producing conditions that would be ‘ideal’ for chondrogenesis while BMP-2 concentrations would be highest at the opposite end, ‘ideal’ for bone. This strategy, therefore, enabled the scaffolds (multilayered fibroin discs glued together with fibrin and implanted as a plug) to mimic conditions within osteochondral tissue [18]. BMP-2 is known to have superior potentiality for osteogenesis than BMP-4 in repairing subchondral bone *in vivo* [40]. The concentrations of BMP-2 (0.1 µg/µL) and TGF-β3 (3 ng/µL) per scaffold disc selected for use here were identified based upon the literature for different scaffold types [41], [42] and after optimizing the maximum volume (3 µL) that could be absorbed using a dye by the test scaffolds. The present study thus also served to underscore the significance of growth factors in the formation and functional integration of neo-osteochondral constructs within the skeletal defects.

Whilst all the *in vivo* constructs examined in this study showed good neo-tissue and host tissue integration, the extent of osteochondral tissue ECM formation not only differed amongst the experimental and control constructs but also between mulberry and non-mulberry silk constructs. All silk scaffold constructs revealed visually indistinguishable dense cellular networks with an intact and visible scaffold lattice. The non-homogenous histological and immunohistochemical staining was indicative of incomplete chondrogenesis [30]. This may be due to relative short duration of *in vivo* culture. As the aim of the current study was to evaluate the osteochondral reconstruction potentiality of two different silk types, the 8 weeks selected was deemed adequate to interpret the differences based on histology and immunohistochemistry. Substantial differences in the type of ECM formed were observed between the two silk scaffold types (*mulberry* vs. *non-mulberry*) and with or without the addition of growth factors. Mulberry scaffold constructs revealed formation of numerous blood vessels enclosed with endothelial cells towards the base while non-mulberry scaffold constructs exhibited fewer blood vessels within the scaffolds and larger chondrocyte like cells at the base indicating possible endochondral ossification. The ECM of mulberry constructs was mainly collagenous (type I collagen in particular) whereas the ECM of non-mulberry constructs was a mix of proteoglycans and collagens (type I and type II collagen). Cumulative findings of the data suggest the ability of silk fibroin to regenerate an osteochondral defect in rat knee joints using a cell free approach with mulberry silk scaffolds differentially favoring osseous tissue formation and non-mulberry silk scaffolds differentially favoring chondral tissue formation after 8 weeks *in vivo* culture.

Osteochondral defects are complex to treat due to the nature of two very different tissues involved. Bone is a highly metabolically active and vascular tissue whereas cartilage is an avascular bradytrophic tissue. Silk fibroin's ability to vascularize and home cells *in vivo* is well established [25], [43], [44]. However, interestingly in the present study, the cell free silk scaffolds, irrespective of whether mulberry or non-mulberry (with/without GFs) could uniformly home surrounding host cells even into the interior of the scaffolds' lattice within an osteochondral defect unlike previous reports, where cell free scaffold-host interaction was depicted as weak and few cells infiltrated at the centre of the defects [43]. The cellular infiltration *in vivo* with coaxed osteochondral differentiation may be from complex cues provided in combination with the physical interaction of the construct with specific endogenous niche that help to mediate functional tissue engineering. The results from this study demonstrated the feasibility of using silk scaffolds from two different species as a 3D implantable platform in osteochondral therapeutics.

## Conclusions

We believe this to be the first report to show that following *in vivo* implantation into OCDs, acellular mulberry and non-mulberry silk scaffolds possess an inherent ability to attract endogenous, joint-resident cells capable of differentially differentiating down the osteo/chondral lineages. The main findings of this study was that silk fibroin scaffolds of *Antheraea mylitta* are more chondroinductive while those of *Bombyx mori* are more osteoinductive when compared under similar conditions, indicating the potential of using a multi-layered combination of these two different kinds of silk fibroin scaffolds for osteochondral defect regeneration with good integration, the stem cell-silk biomaterial interaction, which is exploited in the context of bone or cartilage promoting niche in this investigation, can also be used for other paradigms of tissue engineering to open up new platforms in regenerative medicine.
